# Distribution of industry-sponsored research payments to US physician principal investigators

**DOI:** 10.1371/journal.pone.0358886

**Published:** 2026-09-25

**Authors:** Zhuo Tony Su, Zeyad Hammadeh, Logan B. Galansky, Alyssa G. Arbuiso, Yuezhou Jing, Bruce J. Trock, Misop Han

**Affiliations:** The James Buchanan Brady Urological Institute, Johns Hopkins University School of Medicine, Baltimore, Maryland, United States of America; Gotham Research Ltd, UNITED KINGDOM OF GREAT BRITAIN AND NORTHERN IRELAND

## Abstract

**Background:**

The Open Payments program (OPP) was launched in 2013 following the passage of the Physician Payments Sunshine Act (PPSA) to promote transparency of physician-industry financial relationships. Extensive research has scrutinized general (non-research) payments from industry to physicians. However, industry-sponsored research payments (ISRPs), the largest payment category by value in the OPP, are relatively understudied. We characterized the distribution of ISRPs to US physician principal investigators (PIs) across medical specialties.

**Methods:**

In this retrospective, observational study, we used publicly-available data from the OPP database to identify cash-or-equivalent ISRPs to physician PIs during 2016–2022. We stratified ISRPs by recipient types (to physician PIs directly and indirectly via teaching hospitals and other organizations referred to as non-covered entities [NCEs]). Our primary outcome measures included ISRP distributions by medical specialties of physician PIs. Secondary measures of interest included identifying medical products associated with the highest total payment amounts.

**Results:**

During 2016–2022, ISRPs to physician PIs totaled $33.9 billion, with most ($26.6 billion, 78.5%) directed to physician PIs via NCEs, rather than teaching hospitals or directly to physicians. Physician PIs receiving the highest total payment amounts were general internists, hematologist-oncologists, and neurologists. Anti-cancer drugs, particularly immunotherapy agents, accounted for most of the 25 products associated with the largest payment amounts.

**Conclusions:**

This study revealed significant industry payments in physician-led research, particularly in oncology. Furthermore, the study highlights a need for improved research payment disclosures. OPP reporting rules do not require specific disclosure of payments for research expenses versus physician compensations within an ISRP, nor specific products investigated in industry-sponsored research of experimental products. Improved disclosures will promote transparency of physician-industry financial relationships as originally intended by the PPSA.

## Introduction

After the passage of the Physician Payments Sunshine Act (PPSA) as part of the Affordable Care Act, the Centers for Medicare and Medicaid Services (CMS) launched the Open Payments program (OPP) in 2013 [1]. The OPP is a federally mandated program that requires reporting and publishing of industry payments to physicians and teaching hospitals, with the aim of promoting transparency of physician-industry financial relationships [1–4]. Extensive research has scrutinized *general* (non-research) payments from industry to physicians, such as consulting and speaking fees, travel expenses, gifts, and ownership and investment interests, as these payments may influence physicians’ clinical decision making and affect patients’ trust in healthcare professionals [2–7]. However, industry-sponsored *research* payments (ISRPs) to physicians are relatively understudied, even though these payments constitute the largest payment category by value in the OPP [5, 8]. Existing analyses of ISRPs to physicians have focused on specific, individual specialties [9–11]. There remain limited data regarding the distribution of ISRPs by physician specialties and by medical product types. In this study, we aimed to characterize the distribution of ISRPs to US physician principal investigators (PIs) across specialties and to identify medical products associated with the largest payment amounts.

## Methods

### Study design

This retrospective, observational study follows the Strengthening the Reporting of Observational Studies in Epidemiology (STROBE) guidelines. Institutional review board approval was not applicable because the study used publicly available data.

### Data sources

We accessed OPP data [12] from January 2016 to December 2022 by downloading the data from the program website directly; next, we identified ISRPs (cash and noncash equivalents only) made to US allopathic and osteopathic physician PIs directly, and to teaching hospitals (referred to as covered entities in the OPP) and other organizations (referred to as non-covered entities [NCEs] in the OPP) with physician PIs. We did not include OPP data before 2016 because previous OPP reporting rules during 2013–2015 did not require reporting of the medical specialty of a physician PI and/or medical products involved in industry-sponsored research, precluding stratification of ISRPs by physician specialty and/or product type for those years. We linked physician National Provider Identifiers (NPIs) in the OPP data to the National Plan and Provider Enumeration System (NPPES) [13] and assigned specialties for physician PIs according to the Health Care Provider Taxonomy in the NPPES; 38 specialties were defined (see S1 Table for definitions used to group specialties). If multiple physician PIs were listed for an ISRP (up to five PIs could be listed per ISRP reported to the OPP), we attributed that ISRP exclusively to the primary PI.

### Outcomes

As the primary outcomes, we evaluated ISRPs to physician PIs stratified by physician specialties.

Further, as secondary outcomes, we stratified ISRPs to physician PIs by medical products investigated in industry-sponsored research, specifically by covered versus non-covered product status and product type. In the OPP, a covered product refers to a medical product or service rendered using the product that is reimbursed by Medicare, Medicaid, or Children’s Health Insurance Program. Additionally, if the product is a pharmaceutical, it requires a prescription or a physician’s authorization for administration, or if the product is a device or medical supply, it requires premarket approval or premarket notification by the Food and Drug Administration [12]. Medical products not meeting these criteria are defined as non-covered products in the OPP.

Lastly, we identified 25 manufacturers and 25 medical products associated with the highest total ISRP amounts to physician PIs during 2016–2022. All payment values were inflation-adjusted to 2022 US dollars [14]. We performed data manipulations using Microsoft Excel (Microsoft Corporation, Redmond, Washington) analyses using STATA version 18.0 (StataCorp LLC, College Station, Texas).

## Results

**Fig 1:**
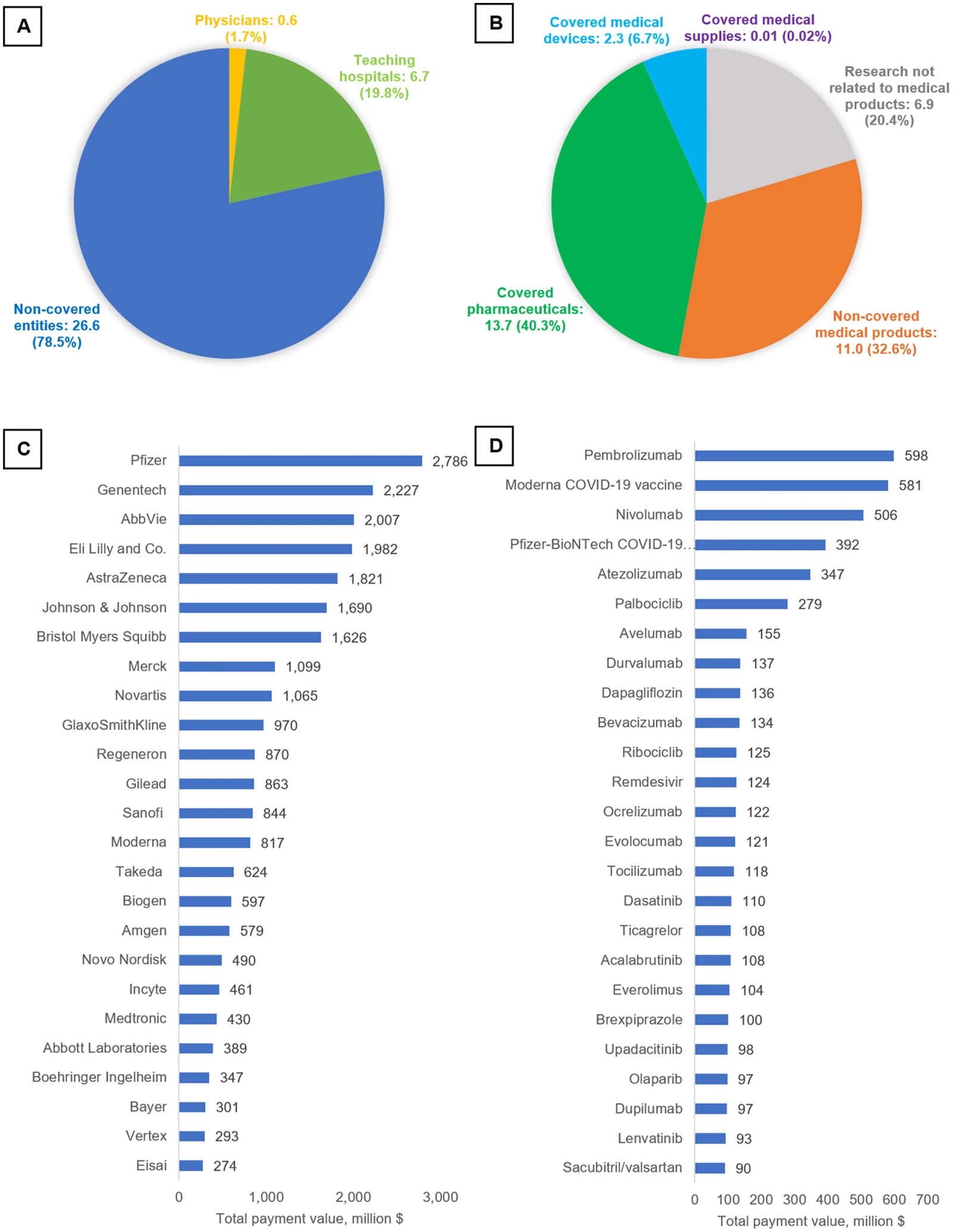
Distribution of industry-sponsored research payments to physician principal investigators in the United States, 2016–2022. Payments, in billion $, stratified by (A) recipient type and by (B) medical product type studied in industry-sponsored research, and the (C) 25 manufacturers and (D) 25 medical products associated with the highest total payment amounts, in million $. Abbreviation: COVID-19, coronavirus disease 2019.

During 2016–2022, a total of 4,132,788 cash-or-equivalent ISRPs with a cumulative value of $33.9 billion were made to 58,653 US physician PIs, including $592 million (1.7%) to physician PIs directly, $6.7 billion (19.8%) to teaching hospitals with physician PIs, and $26.6 billion (78.5%) to NCEs with physician PIs (Fig 1A). Of the $33.9 billion, $27.0 billion (79.6%) was paid to sponsor research of medical products, including $16.0 billion (47.1%) for covered products and $11.0 billion (32.6%) for non-covered products. Among covered products, pharmaceuticals accounted for $13.7 billion of the $16.0 billion (85.7%), medical devices for $2.3 billion (14.2%), and medical supplies for $7.9 million (0.05%; Fig 1B). For ISRPs to physician PIs sponsoring research of non-covered products, product type and name were not disclosed in most of these payment reports, precluding stratification by product type.

Physician PIs receiving the highest total ISRP amounts during 2016–2022 were general internists ($9.0 billion), hematologist-oncologists ($6.2 billion), neurologists ($2.2 billion), cardiologists ($2.1 billion), and psychiatrists ($1.6 billion). Physician PIs with the highest median per-physician payment amounts were hematologist-oncologists ($172,010), infectious disease specialists ($138,203), allergist-immunologist-rheumatologists ($116,637), dermatologists ($115,287), and psychiatrists ($113,954) (Table 1).

**Table 1:** Industry-sponsored research payments (ISRPs) to physician principal investigators (PIs) in the United States, overall and by specialty, from 2016 to 2022.

Specialty of primary PI*	Physician PIs receiving ISRPs, No. (% of overall)	Total amount paid, million $ (% of overall)	Median payment value per physician PI, $ (interquartile range)
General internal medicine	9,860 (16.8)	9,045.9 (26.7)	99,585 (16,956–522,193)
Hematology and oncology	6,446 (11.0)	6,227.3 (18.4)	172,010 (31,872–794,628)
Neurology	3,750 (6.4)	2,243.2 (6.6)	90,720 (20,272–381,100)
Cardiology	5,760 (9.8)	2,115.1 (6.2)	70,706 (18,421–245,082)
Psychiatry	1,304 (2.2)	1,619.1 (4.8)	113,954 (12,381–731,137)
Allergy, immunology, and rheumatology	1,795 (3.1)	1,270.3 (3.7)	116,637 (20,450–509,258)
Gastroenterology	2,152 (3.7)	1,077.5 (3.2)	87,115 (22,608–358,308)
Ophthalmology	1,955 (3.3)	961.1 (2.8)	90,523 (17,978–366,624)
Dermatology	1,245 (2.1)	933.7 (2.8)	115,287 (16,809–546,635)
Pediatrics	2,276 (3.9)	928.9 (2.7)	59,592 (15,738–225,571)
Infectious disease	1,148 (2.0)	899.7 (2.7)	138,203 (27,372–551,229)
Obstetrics and gynecology	2,167 (3.7)	896.3 (2.6)	56,284 (8,575–274,465)
Endocrinology	1,238 (2.1)	793.0 (2.3)	93,619 (20,204–429,114)
Anesthesiology and critical care	1,809 (3.1)	559.2 (1.6)	43,197 (8,013–186,530)
Pulmonology	1,197 (2.0)	549.6 (1.6)	82,102 (16,498–335,181)
General surgery	1,512 (2.6)	538.3 (1.6)	22,660 (3,397–90,585)
Nephrology	1,223 (2.1)	510.2 (1.5)	102,079 (23,141–354,129)
Emergency medicine	698 (1.2)	453.7 (1.3)	61,538 (12,373–307,922)
Orthopedic surgery	2,280 (3.9)	321.7 (0.9)	33,244 (9,769–107,349)
Diagnostic radiology	1,559 (2.7)	305.1 (0.9)	33,802 (8,670–141,283)
Urology	1,031 (1.8)	295.7 (0.9)	43,997 (11,510–165,571)
Neurosurgery	983 (1.7)	202.9 (0.6)	39,682 (10,967–125,224)
Cardiothoracic surgery	758 (1.3)	179.3 (0.5)	51,587 (9,074–217,073)
Pathology	552 (0.9)	172.6 (0.5)	75,063 (15,337–278,573)
Other	206 (0.4)	144.4 (0.4)	92,192 (20,051–526,481)
Otolaryngology	700 (1.2)	141.8 (0.4)	36,325 (9,255–111,247)
Radiation oncology	493 (0.8)	126.1 (0.4)	54,428 (16,385–220,883)
Vascular surgery	538 (0.9)	64.6 (0.2)	38,673 (11,124–113,508)
Preventive medicine	77 (0.1)	55.3 (0.2)	67,956 (11,081–362,089)
Physical medicine and rehabilitation	333 (0.6)	55.0 (0.2)	28,460 (4,888–116,936)
Neuromusculoskeletal and sports medicine	68 (0.1)	54.7 (0.2)	23,871 (6,508–191,971)
Plastic surgery	762 (1.3)	44.2 (0.1)	2,982 (223–27,417)
Transplant surgery	203 (0.3)	40.2 (0.1)	41,496 (11,211–235,191)
Surgical oncology	135 (0.2)	33.5 (0.1)	33,239 (8,200–218,478)
Interventional radiology	250 (0.4)	32.9 (0.1)	33,118 (10,031–128,512)
Colorectal surgery	112 (0.2)	6.6 (0.02)	23,173 (2,171–61,695)
Pediatric surgery	44 (0.1)	6.1 (0.02)	16,849 (9,231–46,362)
Trauma surgery	34 (0.1)	4.2 (0.01)	46,371 (13,433–175,000)
**All physicians receiving ISRPs as a primary PI**	**58,653**	**33,909.2**	**72,029 (14,963–326,772)**

*Note:* All payment values were inflation-adjusted to 2022 United States dollars.

*See **S1 Table** for definitions used to define physician specialties

The manufacturers with the highest total payment amounts during 2016–2022 were Pfizer ($2.8 billion), Genentech ($2.2 billion), AbbVie ($2.0 billion), Eli Lilly and Company ($2.0 billion), and AstraZeneca ($1.8 billion). Among the top 25 manufacturers, most were pharmaceutical and biotechnology companies, and only three—Johnson & Johnson (through its medical device subsidiaries), Medtronic, and Abbott Laboratories—were device manufacturers (Fig 1C). The top 10 manufacturers combined accounted for 50.9% of all ISRPs to physician PIs by value.

The products associated with the highest total payment amounts during 2016–2022 were pembrolizumab ($598 million), Moderna coronavirus disease (COVID-19) vaccine ($581 million), nivolumab ($506 million), Pfizer-BioNTech COVID-19 vaccine ($392 million), and atezolizumab ($347 million). All of the top 25 products were pharmaceuticals. More than half [13] were anti-cancer drugs, including 5 immunotherapy agents (i.e., pembrolizumab, nivolumab, atezolizumab, avelumab, and durvalumab, all ranked among the top 8 products), and 8 targeted therapy agents (Fig 1D).

## Discussion

Our study results demonstrate significant industry payments in physician-led research. During 2016–2022, US physician PIs received $33.9 billion in cash-or-equivalent ISRPs, much higher than non-research payments from industry to physicians. A recent analysis using the OPP data showed that US physicians received $12.1 billion in general (non-research) payments from industry during the period of 2013–2022, including consulting fees, speaking fees, food and beverages, travels and lodging, entertainment, education, gifts, grants, charitable contributions, and honoraria [7].

The OPP was created under the PPSA to bring greater transparency to physician-industry financial relationships that may affect the quality of patient care and integrity of medical research [1, 15]. Through public disclosure of financial relationships, the OPP aims to improve patient awareness of industry payments and influence physician behaviors [1, 15, 16]. The OPP keeps reporting and public disclosure of research payments separate from other payments, recognizing the inherent value of research to society [1, 15]. However, our analysis highlights a clear need for improved disclosure of ISRPs. Currently, there is no required disclosure of fund allocations within an ISRP, particularly regarding payments for direct research expenses versus financial compensations to individual physicians for research activities. Instead, only the research entity receiving a payment, affiliated physician investigators (up to five investigators could be listed per research payment), and the total value of the research payment are mandated for reporting [12, 17]. This limited disclosure can cause confusion for patients and the public in interpreting research payments attributed to physicians. In particular, for physician PIs leading large industry-sponsored clinical trials, total research payment amounts might seem excessive to the lay public. Attributing such large payment values to individual physicians may lead to misconceptions by patients about these physicians’ financial ties to the industry, as the research payment values can vastly exceed any general payments received by those physicians. Such misconceptions may have the unintended consequence of discouraging some physicians from participating in research. On the other hand, if a physician receives direct payment as compensation for participating in industry-sponsored research, clear reporting and disclosure of the amount of the compensation are warranted as originally intended by the PPSA.

Our study also reveals variations in receipt of ISRPs by physician PIs across medical specialties. For example, physicians in general internal medicine and medical subspecialties tend to receive more ISRPs overall and on a per-physician basis than physicians in surgical and procedural-oriented specialties. In particular, hematologist-oncologists and infectious disease specialists received the highest ISRP amounts per physician PI during the analysis period. This likely reflects much higher industry investments in research of pharmaceuticals than devices. Indeed, we have observed that pharmaceuticals accounted for over 85% of payments by value for research of covered medical products, whereas medical devices represented less than 15%. In particular, anti-cancer drugs, including immunotherapy and targeted therapy agents, made up more than half of the top 25 products in total ISRP amounts, reflective of significant research payments in oncology from the pharmaceutical industry. Meanwhile, two of the top four products were COVID-19 vaccines, highlighting substantial resources shifted towards vaccine development and testing during the pandemic.

Our study has several limitations. Firstly, our result accuracies are limited by potential OPP data reporting errors. However, with the enforcement of significant monetary penalties for noncompliance and incomplete/inaccurate disclosure, OPP reporting is expected to be robust [18]. Secondly, because of a lack of disclosure about fund allocation within an ISRP as described above, we had to attribute the entire amount of an ISRP exclusively to the primary PI, to avoid double counting of the same ISRP across multiple physician PIs. This has likely led to misattribution of some research payments to and away from certain physician specialties. Thirdly, our analysis has excluded non-physician PIs, as reporting of payments to non-physician healthcare professionals only began in 2021 [19, 20]. Further, to protect the intellectual property of manufacturers, OPP rules allow manufacturers to request a delay of up to four years in the public disclosure of ISRPs for research and clinical investigations of new medical products or new applications of existing products [12]. Therefore, our reported ISRPs are underestimates for the period of 2019–2022. Additionally, any outdated, incorrect, or incomplete physician specialty data in the NPPES could have contributed to incorrect attribution of ISRPs to physician specialties. Lastly, our analysis of research payments by product type has been limited to covered medical products, as there is no required medical product disclosure in reporting ISRPs for research of non-covered products [12]. This is despite our finding that payments for research of non-covered medical products accounted for about a third of overall ISRPs to physician PIs by value. Therefore, disclosing non-covered medical products (i.e., mostly experimental treatments) investigated in industry-sponsored, physician-led research—after any applicable delay protecting confidentiality during the product development and testing process—represents another area for potential improvement in the reporting and disclosure of ISRPs.

Despite these limitations, our study is the first to characterize the distribution of ISRPs to US physician PIs across medical specialties. Our results demonstrate significant industry payments in physician-led research, especially in active areas including immuno-oncology and targeted therapies, and vaccine development and testing during the COVID-19 pandemic. Moreover, our study highlights a clear need for greater transparency in reporting and public disclosure of ISRPs to physician PIs, including detailed reporting of financial compensation directed towards individual physicians for participating in industry-sponsored research activities, and disclosing specific products tested in industry-sponsored research of experimental medical products. Improved transparency in research payment reporting will help promote the integrity of industry-sponsored medical research, as originally intended by the PPSA.

## Supporting information

S1 Table Taxonomic codes for physician specialties from the Centers for Medicare and Medicaid Services (CMS) Open Payments covered recipient taxonomy list and definitions used to group specialties. (DOCX)
