# Supplementary material for: Distribution of industry-sponsored research payments to US physician principal investigators
Source: PLoS One. 2026 Sep 25;21(9):e0358886. doi: 10.1371/journal.pone.0358886 (PMC13614529; doi:10.1371/journal.pone.0358886)
Supplement: Supplementary file 1 [file pone.0358886.s001.docx]

**S1 Table.** Taxonomic codes for physician specialties from the Centers for Medicare and Medicaid Services (CMS) Open Payments covered recipient taxonomy list and definitions used to group specialties

| Provider taxonomy code | Classification | Specialization | Specialty assigned |
| --- | --- | --- | --- |
| 207LA0401X | Anesthesiology | Addiction Medicine | Anesthesiology and critical care |
| 207LC0200X | Anesthesiology | Critical Care Medicine | Anesthesiology and critical care |
| 207LH0002X | Anesthesiology | Hospice and Palliative Medicine | Anesthesiology and critical care |
| 207LP2900X | Anesthesiology | Pain Medicine | Anesthesiology and critical care |
| 207LP3000X | Anesthesiology | Pediatric Anesthesiology | Anesthesiology and critical care |
| 207L00000X | Anesthesiology |  | Anesthesiology and critical care |
| 207RC0200X | Internal Medicine | Critical Care Medicine | Anesthesiology and critical care |
| 208VP0014X | Pain Medicine | Interventional Pain Medicine | Anesthesiology and critical care |
| 208VP0000X | Pain Medicine | Pain Medicine | Anesthesiology and critical care |
| 2080P0203X | Pediatrics | Pediatric Critical Care Medicine | Anesthesiology and critical care |
| 207RA0002X | Internal Medicine | Adult Congenital Heart Disease | Cardiology |
| 207RA0001X | Internal Medicine | Advanced Heart Failure and Transplant Cardiology | Cardiology |
| 207RC0000X | Internal Medicine | Cardiovascular Disease | Cardiology |
| 207RC0001X | Internal Medicine | Clinical Cardiac Electrophysiology | Cardiology |
| 207RI0011X | Internal Medicine | Interventional Cardiology | Cardiology |
| 2080P0202X | Pediatrics | Pediatric Cardiology | Cardiology |
| 208G00000X | Thoracic Surgery (Cardiothoracic Vascular Surgery) |  | Cardiothoracic surgery |
| 208C00000X | Colon & Rectal Surgery |  | Colorectal surgery |
| 207NI0002X | Dermatology | Clinical & Laboratory Dermatological Immunology | Dermatology |
| 207ND0900X | Dermatology | Dermatopathology | Dermatology |
| 207ND0101X | Dermatology | MOHS-Micrographic Surgery | Dermatology |
| 207NP0225X | Dermatology | Pediatric Dermatology | Dermatology |
| 207NS0135X | Dermatology | Procedural Dermatology | Dermatology |
| 207N00000X | Dermatology |  | Dermatology |
| 207RM1200X | Internal Medicine | Magnetic Resonance Imaging (MRI) | Diagnostic radiology |
| 207UN0903X | Nuclear Medicine | In Vivo & In Vitro Nuclear Medicine | Diagnostic radiology |
| 207UN0901X | Nuclear Medicine | Nuclear Cardiology | Diagnostic radiology |
| 207UN0902X | Nuclear Medicine | Nuclear Imaging & Therapy | Diagnostic radiology |
| 207U00000X | Nuclear Medicine |  | Diagnostic radiology |
| 2084D0003X | Psychiatry & Neurology | Diagnostic Neuroimaging | Diagnostic radiology |
| 2085B0100X | Radiology | Body Imaging | Diagnostic radiology |
| 2085D0003X | Radiology | Diagnostic Neuroimaging | Diagnostic radiology |
| 2085R0202X | Radiology | Diagnostic Radiology | Diagnostic radiology |
| 2085U0001X | Radiology | Diagnostic Ultrasound | Diagnostic radiology |
| 2085H0002X | Radiology | Hospice and Palliative Medicine | Diagnostic radiology |
| 2085N0700X | Radiology | Neuroradiology | Diagnostic radiology |
| 2085N0904X | Radiology | Nuclear Radiology | Diagnostic radiology |
| 2085P0229X | Radiology | Pediatric Radiology | Diagnostic radiology |
| 2085R0205X | Radiology | Radiological Physics | Diagnostic radiology |
| 207PE0004X | Emergency Medicine | Emergency Medical Services | Emergency medicine |
| 207PH0002X | Emergency Medicine | Hospice and Palliative Medicine | Emergency medicine |
| 207PT0002X | Emergency Medicine | Medical Toxicology | Emergency medicine |
| 207PP0204X | Emergency Medicine | Pediatric Emergency Medicine | Emergency medicine |
| 207PS0010X | Emergency Medicine | Sports Medicine | Emergency medicine |
| 207PE0005X | Emergency Medicine | Undersea and Hyperbaric Medicine | Emergency medicine |
| 207P00000X | Emergency Medicine |  | Emergency medicine |
| 2080P0204X | Pediatrics | Pediatric Emergency Medicine | Emergency medicine |
| 207RE0101X | Internal Medicine | Endocrinology, Diabetes & Metabolism | Endocrinology |
| 2080P0205X | Pediatrics | Pediatric Endocrinology | Endocrinology |
| 207RG0100X | Internal Medicine | Gastroenterology | Gastroenterology |
| 207RI0008X | Internal Medicine | Hepatology | Gastroenterology |
| 207RT0003X | Internal Medicine | Transplant Hepatology | Gastroenterology |
| 2080P0206X | Pediatrics | Pediatric Gastroenterology | Gastroenterology |
| 2080T0004X | Pediatrics | Pediatric Transplant Hepatology | Gastroenterology |
| 207QA0505X | Family Medicine | Adult Medicine | General internal medicine |
| 207QG0300X | Family Medicine | Geriatric Medicine | General internal medicine |
| 207QH0002X | Family Medicine | Hospice and Palliative Medicine | General internal medicine |
| 207QB0002X | Family Medicine | Obesity Medicine | General internal medicine |
| 207QS1201X | Family Medicine | Sleep Medicine | General internal medicine |
| 207Q00000X | Family Medicine |  | General internal medicine |
| 208D00000X | General Practice |  | General internal medicine |
| 208M00000X | Hospitalist |  | General internal medicine |
| 207RG0300X | Internal Medicine | Geriatric Medicine | General internal medicine |
| 207RH0002X | Internal Medicine | Hospice and Palliative Medicine | General internal medicine |
| 207RH0005X | Internal Medicine | Hypertension Specialist | General internal medicine |
| 207RB0002X | Internal Medicine | Obesity Medicine | General internal medicine |
| 207RS0012X | Internal Medicine | Sleep Medicine | General internal medicine |
| 207R00000X | Internal Medicine |  | General internal medicine |
| 2086H0002X | Surgery | Hospice and Palliative Medicine | General surgery |
| 2086S0102X | Surgery | Surgical Critical Care | General surgery |
| 208600000X | Surgery |  | General surgery |
| 207RH0000X | Internal Medicine | Hematology | Hematology and oncology |
| 207RH0003X | Internal Medicine | Hematology & Oncology | Hematology and oncology |
| 207RX0202X | Internal Medicine | Medical Oncology | Hematology and oncology |
| 2080P0207X | Pediatrics | Pediatric Hematology-Oncology | Hematology and oncology |
| 207RI0200X | Internal Medicine | Infectious Disease | Infectious disease |
| 2080P0208X | Pediatrics | Pediatric Infectious Diseases | Infectious disease |
| 2085R0204X | Radiology | Vascular & Interventional Radiology | Interventional radiology |
| 207RN0300X | Internal Medicine | Nephrology | Nephrology |
| 2080P0210X | Pediatrics | Pediatric Nephrology | Nephrology |
| 204R00000X | Electrodiagnostic Medicine |  | Neurology |
| 2080P0008X | Pediatrics | Neurodevelopmental Disabilities | Neurology |
| 2084B0040X | Psychiatry & Neurology | Behavioral Neurology & Neuropsychiatry | Neurology |
| 2084P0301X | Psychiatry & Neurology | Brain Injury Medicine | Neurology |
| 2084N0600X | Psychiatry & Neurology | Clinical Neurophysiology | Neurology |
| 2084E0001X | Psychiatry & Neurology | Epilepsy | Neurology |
| 2084H0002X | Psychiatry & Neurology | Hospice and Palliative Medicine | Neurology |
| 2084A2900X | Psychiatry & Neurology | Neurocritical Care | Neurology |
| 2084P0005X | Psychiatry & Neurology | Neurodevelopmental Disabilities | Neurology |
| 2084N0400X | Psychiatry & Neurology | Neurology | Neurology |
| 2084N0402X | Psychiatry & Neurology | Neurology with Special Qualifications in Child Neurology | Neurology |
| 2084N0008X | Psychiatry & Neurology | Neuromuscular Medicine | Neurology |
| 2084P2900X | Psychiatry & Neurology | Pain Medicine | Neurology |
| 2084S0012X | Psychiatry & Neurology | Sleep Medicine | Neurology |
| 2084S0010X | Psychiatry & Neurology | Sports Medicine | Neurology |
| 2084V0102X | Psychiatry & Neurology | Vascular Neurology | Neurology |
| 207QS0010X | Family Medicine | Sports Medicine | Neuromusculoskeletal and sports medicine |
| 207RS0010X | Internal Medicine | Sports Medicine | Neuromusculoskeletal and sports medicine |
| 204D00000X | Neuromusculoskeletal Medicine & OMM |  | Neuromusculoskeletal and sports medicine |
| 204C00000X | Neuromusculoskeletal Medicine, Sports Medicine |  | Neuromusculoskeletal and sports medicine |
| 207T00000X | Neurological Surgery |  | Neurosurgery |
| 207VC0300X | Obstetrics & Gynecology | Complex Family Planning | Obstetrics and gynecology |
| 207VC0200X | Obstetrics & Gynecology | Critical Care Medicine | Obstetrics and gynecology |
| 207VF0040X | Obstetrics & Gynecology | Female Pelvic Medicine and Reconstructive Surgery | Obstetrics and gynecology |
| 207VX0201X | Obstetrics & Gynecology | Gynecologic Oncology | Obstetrics and gynecology |
| 207VG0400X | Obstetrics & Gynecology | Gynecology | Obstetrics and gynecology |
| 207VH0002X | Obstetrics & Gynecology | Hospice and Palliative Medicine | Obstetrics and gynecology |
| 207VM0101X | Obstetrics & Gynecology | Maternal & Fetal Medicine | Obstetrics and gynecology |
| 207VB0002X | Obstetrics & Gynecology | Obesity Medicine | Obstetrics and gynecology |
| 207VX0000X | Obstetrics & Gynecology | Obstetrics | Obstetrics and gynecology |
| 207VE0102X | Obstetrics & Gynecology | Reproductive Endocrinology | Obstetrics and gynecology |
| 207V00000X | Obstetrics & Gynecology |  | Obstetrics and gynecology |
| 207WX0120X | Ophthalmology | Cornea and External Diseases Specialist | Ophthalmology |
| 207WX0009X | Ophthalmology | Glaucoma Specialist | Ophthalmology |
| 207WX0109X | Ophthalmology | Neuro-ophthalmology | Ophthalmology |
| 207WX0200X | Ophthalmology | Ophthalmic Plastic and Reconstructive Surgery | Ophthalmology |
| 207WX0110X | Ophthalmology | Pediatric Ophthalmology and Strabismus Specialist | Ophthalmology |
| 207WX0107X | Ophthalmology | Retina Specialist | Ophthalmology |
| 207WX0108X | Ophthalmology | Uveitis and Ocular Inflammatory Disease | Ophthalmology |
| 207W00000X | Ophthalmology |  | Ophthalmology |
| 207XS0114X | Orthopaedic Surgery | Adult Reconstructive Orthopaedic Surgery | Orthopedic surgery |
| 207XX0004X | Orthopaedic Surgery | Foot and Ankle Surgery | Orthopedic surgery |
| 207XS0106X | Orthopaedic Surgery | Hand Surgery | Orthopedic surgery |
| 207XS0117X | Orthopaedic Surgery | Orthopaedic Surgery of the Spine | Orthopedic surgery |
| 207XX0801X | Orthopaedic Surgery | Orthopaedic Trauma | Orthopedic surgery |
| 207XP3100X | Orthopaedic Surgery | Pediatric Orthopaedic Surgery | Orthopedic surgery |
| 207XX0005X | Orthopaedic Surgery | Sports Medicine | Orthopedic surgery |
| 207X00000X | Orthopaedic Surgery |  | Orthopedic surgery |
| 208U00000X | Clinical Pharmacology |  | Other |
| 202C00000X | Independent Medical Examiner |  | Other |
| 202D00000X | Integrative Medicine |  | Other |
| 209800000X | Legal Medicine |  | Other |
| 207SG0202X | Medical Genetics | Clinical Biochemical Genetics | Other |
| 207SC0300X | Medical Genetics | Clinical Cytogenetic | Other |
| 207SG0201X | Medical Genetics | Clinical Genetics (M.D.) | Other |
| 207SG0203X | Medical Genetics | Clinical Molecular Genetics | Other |
| 207SG0205X | Medical Genetics | Ph.D. Medical Genetics | Other |
| 204E00000X | Oral & Maxillofacial Surgery |  | Other |
| 202K00000X | Phlebology |  | Other |
| 207YS0123X | Otolaryngology | Facial Plastic Surgery | Otolaryngology |
| 207YX0602X | Otolaryngology | Otolaryngic Allergy | Otolaryngology |
| 207YX0905X | Otolaryngology | Otolaryngology/Facial Plastic Surgery | Otolaryngology |
| 207YX0901X | Otolaryngology | Otology & Neurotology | Otolaryngology |
| 207YP0228X | Otolaryngology | Pediatric Otolaryngology | Otolaryngology |
| 207YX0007X | Otolaryngology | Plastic Surgery within the Head & Neck | Otolaryngology |
| 207YS0012X | Otolaryngology | Sleep Medicine | Otolaryngology |
| 207Y00000X | Otolaryngology |  | Otolaryngology |
| 207SM0001X | Medical Genetics | Molecular Genetic Pathology | Pathology |
| 207ZP0101X | Pathology | Anatomic Pathology | Pathology |
| 207ZP0102X | Pathology | Anatomic Pathology & Clinical Pathology | Pathology |
| 207ZB0001X | Pathology | Blood Banking & Transfusion Medicine | Pathology |
| 207ZP0104X | Pathology | Chemical Pathology | Pathology |
| 207ZC0008X | Pathology | Clinical Informatics | Pathology |
| 207ZC0006X | Pathology | Clinical Pathology | Pathology |
| 207ZP0105X | Pathology | Clinical Pathology/Laboratory Medicine | Pathology |
| 207ZC0500X | Pathology | Cytopathology | Pathology |
| 207ZD0900X | Pathology | Dermatopathology | Pathology |
| 207ZF0201X | Pathology | Forensic Pathology | Pathology |
| 207ZH0000X | Pathology | Hematology | Pathology |
| 207ZI0100X | Pathology | Immunopathology | Pathology |
| 207ZM0300X | Pathology | Medical Microbiology | Pathology |
| 207ZP0007X | Pathology | Molecular Genetic Pathology | Pathology |
| 207ZN0500X | Pathology | Neuropathology | Pathology |
| 207ZP0213X | Pathology | Pediatric Pathology | Pathology |
| 2086S0120X | Surgery | Pediatric Surgery | Pediatric surgery |
| 207QA0000X | Family Medicine | Adolescent Medicine | Pediatrics |
| 207RA0000X | Internal Medicine | Adolescent Medicine | Pediatrics |
| 2080A0000X | Pediatrics | Adolescent Medicine | Pediatrics |
| 2080C0008X | Pediatrics | Child Abuse Pediatrics | Pediatrics |
| 2080P0006X | Pediatrics | Developmental – Behavioral Pediatrics | Pediatrics |
| 2080H0002X | Pediatrics | Hospice and Palliative Medicine | Pediatrics |
| 2080T0002X | Pediatrics | Medical Toxicology | Pediatrics |
| 2080N0001X | Pediatrics | Neonatal-Perinatal Medicine | Pediatrics |
| 2080B0002X | Pediatrics | Obesity Medicine | Pediatrics |
| 2080S0012X | Pediatrics | Sleep Medicine | Pediatrics |
| 2080S0010X | Pediatrics | Sports Medicine | Pediatrics |
| 208000000X | Pediatrics |  | Pediatrics |
| 2081P0301X | Physical Medicine & Rehabilitation | Brain Injury Medicine | Physical medicine and rehabilitation |
| 2081H0002X | Physical Medicine & Rehabilitation | Hospice and Palliative Medicine | Physical medicine and rehabilitation |
| 2081N0008X | Physical Medicine & Rehabilitation | Neuromuscular Medicine | Physical medicine and rehabilitation |
| 2081P2900X | Physical Medicine & Rehabilitation | Pain Medicine | Physical medicine and rehabilitation |
| 2081P0010X | Physical Medicine & Rehabilitation | Pediatric Rehabilitation Medicine | Physical medicine and rehabilitation |
| 2081P0004X | Physical Medicine & Rehabilitation | Spinal Cord Injury Medicine | Physical medicine and rehabilitation |
| 2081S0010X | Physical Medicine & Rehabilitation | Sports Medicine | Physical medicine and rehabilitation |
| 208100000X | Physical Medicine & Rehabilitation |  | Physical medicine and rehabilitation |
| 2082S0099X | Plastic Surgery | Plastic Surgery Within the Head and Neck | Plastic surgery |
| 2082S0105X | Plastic Surgery | Surgery of the Hand | Plastic surgery |
| 208200000X | Plastic Surgery |  | Plastic surgery |
| 2086S0122X | Surgery | Plastic and Reconstructive Surgery | Plastic surgery |
| 2086S0105X | Surgery | Surgery of the Hand | Plastic surgery |
| 2083A0300X | Preventive Medicine | Addiction Medicine | Preventive medicine |
| 2083A0100X | Preventive Medicine | Aerospace Medicine | Preventive medicine |
| 2083C0008X | Preventive Medicine | Clinical Informatics | Preventive medicine |
| 2083T0002X | Preventive Medicine | Medical Toxicology | Preventive medicine |
| 2083B0002X | Preventive Medicine | Obesity Medicine | Preventive medicine |
| 2083X0100X | Preventive Medicine | Occupational Medicine | Preventive medicine |
| 2083P0500X | Preventive Medicine | Preventive Medicine/Occupational Environmental Medicine | Preventive medicine |
| 2083P0901X | Preventive Medicine | Public Health & General Preventive Medicine | Preventive medicine |
| 2083S0010X | Preventive Medicine | Sports Medicine | Preventive medicine |
| 2083P0011X | Preventive Medicine | Undersea and Hyperbaric Medicine | Preventive medicine |
| 207QA0401X | Family Medicine | Addiction Medicine | Psychiatry |
| 207RA0401X | Internal Medicine | Addiction Medicine | Psychiatry |
| 2084A0401X | Psychiatry & Neurology | Addiction Medicine | Psychiatry |
| 2084P0802X | Psychiatry & Neurology | Addiction Psychiatry | Psychiatry |
| 2084P0804X | Psychiatry & Neurology | Child & Adolescent Psychiatry | Psychiatry |
| 2084F0202X | Psychiatry & Neurology | Forensic Psychiatry | Psychiatry |
| 2084P0805X | Psychiatry & Neurology | Geriatric Psychiatry | Psychiatry |
| 2084B0002X | Psychiatry & Neurology | Obesity Medicine | Psychiatry |
| 2084P0800X | Psychiatry & Neurology | Psychiatry | Psychiatry |
| 2084P0015X | Psychiatry & Neurology | Psychosomatic Medicine | Psychiatry |
| 207RP1001X | Internal Medicine | Pulmonary Disease | Pulmonology |
| 2080P0214X | Pediatrics | Pediatric Pulmonology | Pulmonology |
| 2085R0001X | Radiology | Radiation Oncology | Radiation oncology |
| 2085R0203X | Radiology | Therapeutic Radiology | Radiation oncology |
| 207KA0200X | Allergy & Immunology | Allergy | Allergy, immunology, and rheumatology |
| 207KI0005X | Allergy & Immunology | Clinical & Laboratory Immunology | Allergy, immunology, and rheumatology |
| 207K00000X | Allergy & Immunology |  | Allergy, immunology, and rheumatology |
| 207RA0201X | Internal Medicine | Allergy & Immunology | Allergy, immunology, and rheumatology |
| 207RI0001X | Internal Medicine | Clinical & Laboratory Immunology | Allergy, immunology, and rheumatology |
| 207RR0500X | Internal Medicine | Rheumatology | Allergy, immunology, and rheumatology |
| 2080I0007X | Pediatrics | Clinical & Laboratory Immunology | Allergy, immunology, and rheumatology |
| 2080P0201X | Pediatrics | Pediatric Allergy/Immunology | Allergy, immunology, and rheumatology |
| 2080P0216X | Pediatrics | Pediatric Rheumatology | Allergy, immunology, and rheumatology |
| 2086X0206X | Surgery | Surgical Oncology | Surgical oncology |
| 204F00000X | Transplant Surgery |  | Transplant surgery |
| 2086S0127X | Surgery | Trauma Surgery | Trauma surgery |
| 2088F0040X | Urology | Female Pelvic Medicine and Reconstructive Surgery | Urology |
| 2088P0231X | Urology | Pediatric Urology | Urology |
| 208800000X | Urology |  | Urology |
| 2086S0129X | Surgery | Vascular Surgery | Vascular surgery |
